# Persistent Infection of Simian Foamy Virus Derived from the Japanese Macaque Leads to the High-Level Expression of microRNA that Resembles the miR-1 microRNA Precursor Family

**DOI:** 10.1264/jsme2.ME19130

**Published:** 2020-01-23

**Authors:** Akira Hashimoto-Gotoh, Koichi Kitao, Takayuki Miyazawa

**Affiliations:** 1 Laboratory of Virus-Host Coevolution, Institute for Frontier Life and Medical Sciences, Kyoto University, Kyoto 606–8507, Japan; 2 International Research Unit of Advanced Future Studies, Kyoto University, Kyoto 606–8502, Japan

**Keywords:** simian foamy virus, microRNA, miR-1 microRNA precursor family, adenylyl cyclase-associated protein 1

## Abstract

MicroRNAs (miRNAs) are a group of small non-coding RNAs that suppress the expression of target mRNAs. The seed sequence of miRNA plays a crucial role in recognizing the 3′-untranslated region of the target mRNA. Cells infected with a simian foamy virus (SFV) isolated from an African green monkey (*Chlorocebus aethiops*) (SFVcae) showed high expression levels of viral miRNAs encoded in the long terminal repeat of SFVcae. In the present study, we investigated the roles and expression of miRNAs derived from an SFV isolated from a Japanese macaque (*Macaca fuscata*) (SFVmfu) using next-generation sequencing technologies. The results obtained showed that SFVmfu also expressed viral miRNAs; however, the seed sequences of most miRNAs derived from SFVmfu differed from those reported previously from SFVcae. Cells persistently infected with SFVmfu strongly expressed an miRNA with the same seed sequence as the miR-1 microRNA precursor family. Luciferase reporter assays indicated that this miRNA down-regulates the expression of adenylyl cyclase-associated protein 1, which is up-regulated in several solid tumors. The present results suggest that SFVmfu utilizes viral miRNAs to establish long-term co-existence with the Japanese macaque.

Simian foamy virus (SFV) is a non-pathogenetic retrovirus that belongs to the genus *Simiispumavirus*, sub-family *Spumaviridae*, family *Retroviridae* (Khan *et al.*, 2018). SFV is commonly observed across the primates (Pinto-Santini *et al.*, 2017). Compared to other retroviruses belonging to the sub-family *Orthoretroviridae*, the mutation rate of foamy viruses (FVs) is extremely low (1.7×10^–8^ substitutions site^–1^ year^–1^) and the phylogenetic tree of SFVs from various host species is congruent with that of the hosts, which indicates that SFV undergoes co-speciation with the host primate species (Switzer *et al.*, 2005; Liu *et al.*, 2008). SFVs and host primates have co-existed for millions of years, which indicates that a symbiotic relationship exists between them.

MicroRNAs (miRNAs) are a group of small non-coding RNAs that are transcribed by RNA polymerase II or III (Abdelfattah *et al.*, 2014). Processed mature miRNAs will be incorporated into a series of proteins, such as the Argonaute, to form the RNA-induced silencing complex (RISC) (Chendrimada *et al.*, 2005). Once incorporated into RISC, one of the miRNA strands (3 prime [p] or 5p) will serve as a “guide” to recognize the complementary sequence present in the 3′-untranslated region (3′UTR) of target mRNA, and the expression of the target gene will be repressed by RISC via deadenylation-mediated decay (Eulalio *et al.*, 2009). The seed sequences of miRNAs (nucleotides at 2–7 or 8 bases from the 5′-terminal) are considered to be the critical component for the miRNA-induced targeting of RISC (Bartel, 2009).

Long terminal repeats (LTRs) are repetitive sequences that are present in both ends of the proviral DNA of retroviruses. LTR is composed of three regions; U3, R, and U5 (Fig. 1B): the U3 region contains the enhancer and promoter elements; the R region starts from the transcription start site (TSS) and contains the polyadenylation signal (Thompson *et al.*, 2016). The lengths of the LTRs (particularly the U3 region) of FVs are longer than those of the other retroviruses. The U3 region of FVs partially overlaps with an ORF (termed ORF-2) encoding an auxiliary gene of FVs (Fig. 1B). A recent study reported that SFV LTR encodes miRNAs, which are expressed upon infection (Kincaid *et al.*, 2014). Not only SFV, but also several retroviruses express miRNAs upon infection. These viruses include bovine leukemia virus, avian leukosis virus, human immunodeficiency virus 1, and bovine FV (Klase *et al.*, 2007; Kincaid *et al.*, 2012; Whisnant *et al.*, 2014; Yao *et al.*, 2014). miRNAs encoded in the LTRs of SFV and bovine FV include unique dumbbell-shaped pre-miRNAs that are derived from transcripts that contain two adjacent pre-miRNAs separated by just a few bases (Kincaid *et al.*, 2014; Whisnant *et al.*, 2014). The dumbbell-shaped miRNAs derived from the LTR U3 region of FVs have been shown to repress the expression of target genes (Cao *et al.*, 2018). Therefore, the miRNAs of FVs are expected to play roles in viral replication and persistent infection; however, the functions of these miRNAs currently remain unclear.

The miRNAs derived from the LTR of SFV isolated from an African green monkey (*Chlorocebus aethiops*) (SFVcae) have been extensively analyzed (Kincaid *et al.*, 2014). However, the LTR is less conserved than the other viral genes in SFVs (Galvin *et al.*, 2013). Distinct SFVs may express miRNAs with different seed sequences that target different genes. We herein performed analyses of the miRNAs of SFV derived from a Japanese macaque (*Macaca fuscata*) (SFVmfu). This virus was previously abbreviated as SFVjm (Yoshikawa *et al.*, 2014), which was later renamed SFVmfu (Khan *et al.*, 2018). We revealed that these miRNAs were also expressed from the LTR of SFVmfu. Nevertheless, the seed sequences of the miRNAs derived from SFVmfu were different from those of SFVcae. Notably, an miRNA that was expressed at the highest level in SFVmfu shared the seed sequence with the miR-1 miRNA precursor family of the host. Transcriptome and immunoblot analyses and reporter assays indicated that adenylyl cyclase-associated protein 1 (CAP1) is one of the targets of the miRNA.

## Materials and Methods

### Cells and viruses

Human embryonic kidney (HEK) 293T (ATCC, CRL-11268) (Graham *et al.*, 1977), TE671 (derived from human rhabdomyosarcoma) (ATCC, HTB-139) (Stratton *et al.*, 1989), and *M. dunni* (Clone III8C) cells (derived from the skin fibroblasts of *Mus terricolor*) (ATCC, CRL-2017) (Lander and Chattopadhyay, 1984) were cultured in Dulbecco’s modified Eagle’s medium (Sigma Aldrich) supplemented with 10% heat-inactivated fetal calf serum, penicillin (100 units mL^–1^), and streptomycin (100 mg mL^–1^) (Invitrogen) at 37°C in a humidified atmosphere of 5% CO_2_ in air. An SFVmfu indicator cell line (*M. dunni* cells encoding the β-galactosidase gene driven by the SFVmfu LTR), termed MD(SFVmfu-*lacZ*) cells, was newly established using the same approach as that described previously (Lambert *et al.*, 2016). MD(SFVmfu-*lacZ*) cells were cultured in the same medium as that described above, except that puromycin (5 μg mL^–1^) was added. To prepare stock viruses of the SFVmfu strain WK1, *M. dunni* cells were transfected with an infectious molecular clone (named pJM356) (Yoshikawa *et al.*, 2014) of the strain using Lipofectamine 2000 (Thermo Fisher Scientific) according to the manufacturer’s instructions. Transfected cells were further cultured until syncytial foci became prominent. Cells were then freeze-thawed three times and sonicated to release intracellular virus particles. Cell debris was removed by centrifugation at 3,000 rpm for 10‍ ‍min, and viruses were collected as stock viruses by filtrating with 450-nm membrane filters (GVS). Stock viruses were stored at –80°C until used.

### Titration of SFVmfu

To titrate stock viruses of SFVmfu, the LacZ assay was performed using MD(SFVmfu-*lacZ*) cells as indicator cells. Briefly, 2×10^4^ of MD(SFVmfu-*lacZ*) cells were plated in each well of a 96-well plate (Thermo Fisher Scientific) 1‍ ‍d before the inoculation. After 1 d, stock viruses were serially diluted and immediately inoculated into these cells under the presence of polybrene (8‍ ‍μg‍ ‍mL‍^–1^), and the inoculated cells were incubated for viral absorption for 4 h at 37°C in a humidified atmosphere of 5% CO_2_ in air. After the inoculation, inocula were replaced with 0.2 mL of fresh medium, and further incubated for an additional 2 d. Cells were then fixed with 1% glutaraldehyde and stained with X-Gal (5-bromo-4-chloro-3-indolyl-β-D-galactopyranoside), and *lacZ*-positive foci were counted for titration as described previously (Sakaguchi *et al.*, 2008; Hashimoto-Gotoh *et al.*, 2015).

### Establishment of TE671 cells persistently infected with SFVmfu

TE671 cells persistently infected with the SFVmfu strain WK1 (clone JM356) were established as described previously (Miyazawa *et al.*, 1995). Briefly, SFVmfu was inoculated into TE671 cells at a multiplicity of infection (MOI) of 0.172. Cells were cultured and passaged several times until severe cytopathic effects (syncytial formation and cell death) were observed. After the majority of cells had detached from the culture plate, surviving cells were co-cultured with uninfected TE671 cells. The co-cultured cells continued to grow, showing mild cytopathic effects. After several passages, cells were able to be passaged regularly (2-4-fold dilution per 4–5 d). We designated these cells as TE671/SFVmfu(PI) cells.

### Construction of plasmids

Genomic DNAs were extracted from uninfected TE671 and TE671/SFVmfu(PI) cells using the QIAamp DNA Blood Mini kit (QIAGEN). Genomic DNAs were used as templates to construct miRNA expression plasmids. All primers used in the present study are listed in Table S1. PCR was performed using KOD-Plus-Neo (Toyobo) according to the manufacturer’s instructions. In PCR, we used 200-μL thin-walled tubes and a C1000 thermal cycler (Bio-Rad Laboratories). Regarding hsa-mir-1, the amplified fragment was cloned into pcDNA3.1(+) digested with *Xba*I/*Xho*I to become pcDNA3.1(+)/hsa-mir-1 (Fig. 3A [top]). The amplified fragment for SFVmfu-mir-S6-7 was cloned into pUC18 digested with *Hin*dIII/*Bam*HI to become pUC18/SFVmfu-mir-S6-7 (Fig. 3A [center]).

A series of firefly luciferase reporter plasmids was constructed with inserts at the 3′-end of firefly luciferase. Briefly, the human cytomegalovirus (hCMV) enhancer/promoter was cloned into the *Nhe*I/*Hin*dIII sites of pGL3-basic (Promega) to become pGL3-hCMV (Fig. 3A [bottom]). The complementary sequence of each mature miRNA (hsa-miR-1 or SFVmfu-miR-S7-5p) was prepared by annealing and extending the synthetic oligo and cloned into the *Xba*I site of the pGL3-hCMV using NEBuilder HiFi DNA Assembly (New England Biolab) to become pGL3-hCMV/hsa-miR-1 or pGL3/hCMV/SFVmfu-miR-S7-5p (Fig. 3A [bottom]). Similarly, the 3′UTR of human CAP1 was cloned into the *Xba*I site of pGL3-hCMV to become pGL3-hCMV/CAP1 3′UTR (Wild-type) (Fig. 4A). Using pGL3-hCMV/CAP1 3′UTR (Wild-type) as a template, site-directed mutagenesis was performed at the hsa-miR-1/SFVmfu-miR-S7-5p recognition sites within CAP1 3′UTR to become pGL3-hCMV/CAP1 3′UTR (Single mutant [mut.]) and pGL3-hCMV/CAP1 3′UTR (Triple mut.) (Fig. 4A). Recognition sites were predicted using the online database miRDB version 6.0 (http://www.mirdb.org/) (Wong and Wang, 2015; Liu and Wang, 2019).

### Next-generation sequencing analysis

Small RNA sequencing and transcriptome analyses were performed on TE671 and TE671/SFVmfu(PI) cells. Total RNAs were extracted from cells using RNAzol (Promega), followed by a DNase I (Roche) treatment without a heat inactivation step, and re-extracted using RNAzol. Re-extracted RNAs were further processed by a commercial next-generation sequencing company (Novogene).

Regarding small RNA sequencing, sequencing libraries were constructed from total RNAs using the NEBNext Multiplex Small RNA Library Prep Set for Illumina (New England Biolab), which generates strand-specific small RNA libraries. Size selection of the cDNA libraries was performed using native polyacrylamide gel (12%)-electrophoresis to isolate a fraction containing miRNA-derived cDNAs. Single-end 50-bp sequencing was then performed on an Illumina Hiseq 2500 platform. Sequencing adapter sequences were trimmed from the pre-processed reads using Trimmomatic (ver. 0.38) followed by Fastp (ver. 0.19.4) (Bolger *et al.*, 2014; Chen *et al.*, 2018). Reads were then mapped to the viral genome of the SFVmfu clone JM356 (Yoshikawa *et al.*, 2014) using Bowtie2 (ver. 2.3.4.3) (Langmead and Salzberg, 2012) and converted to the bam file using Samtools (ver. 1.9) (Li *et al.*, 2009). Bam files were converted to bed files, and the coverage depth was calculated using Bedtools (ver. 2.28.0) (Quinlan and Hall, 2010). The secondary structures of miRNA were predicted using RNAfold with default parameters (Gruber *et al.*, 2008; Lorenz *et al.*, 2011). Coverage depth was visualized using Microsoft Excel (ver. 16.28).

In transcriptome analyses, sequencing libraries were constructed using the NEBNext Ultra RNA Library Prep Kit for Illumina (New England Biolab), which generates non-strand-specific cDNA libraries. Paired-end 150-bp sequencing was performed on an Illumina Hiseq 2500 platform. Reads were mapped to the human genome (GRCh38.p12) using Hisat2 (ver. 2.1.0) (Kim *et al.*, 2015) and converted to a bam file using Samtools (ver. 1.9) (Li *et al.*, 2009). The mapped reads were assembled using Cufflinks (ver. 2.1.1), and expression differences between uninfected TE671 and TE671/SFVmfu(PI) cells were calculated using Cuffmerge (ver. 2.1.1) and Cuffdiff (ver. 2.1.1) (Trapnell *et al.*, 2010; 2012). The potential targets of SFVmfu-miR-S7-5p were predicted using the online database miRDB version 6 (Wong and Wang, 2015; Liu and Wang, 2019). Calculated expression levels were visualized using Microsoft Excel (ver. 16.28).

### Multiple sequencing alignment analyses of CAP1 3′UTRs and SFV LTRs from various species

The genomic DNA of a Japanese macaque was extracted from blood using the QIAamp DNA Blood Mini kit (QIAGEN). Extracted DNA was used as a template to amplify the 3′UTR of CAP1 by PCR using the primers listed in Table S1. The amplified product was cloned into the *Hin*dIII/*Eco*RI sites of pcDNA3.1(+). The reverse primer used for PCR was applied for sequencing. A sequencing analysis was performed by a commercial DNA sequencing company (Fasmac). The 3′UTR sequences of CAP1 from humans (*Homo sapiens*), African green monkeys (AGM), crab-eating macaques (*M. fascicularis*), and rhesus macaques (*M. mulatta*) were retrieved from ENSEMBL release 97 (human: GRCh38.p12, vervet AGM: ChlSab1.1, crab-eating macaque: Macaca_fascicularis_5.0, rhesus macaque: Mmul_8.0.1) (Accessed in July 2019).

The LTR sequences of the following SFVs were retrieved from the National Center of Biotechnology Information: SFVcae_LK3 (NC_010820), SFVmcy_FV34 (KF026286.1), SFVmmu_DPZ9524 (MG051205.1) and SFVmfu_WK1.pJM356 (NC_039026.1), which are derived from AGM, Taiwanese macaques (*M. cyclopis*), rhesus macaques, and Japanese macaques, respectively.

Multiple sequence alignment analyses of CAP1 3′UTRs and SFV LTRs were performed using the MAFFT program (ver. 7.407) with the L-INS-i method (Katoh and Standley, 2013). The aligned sequences were visualized using Seaview (ver. 4.7) (Gouy *et al.*, 2010).

### Immunoblot analyses

Whole cell lysates of TE671 cells were obtained with RIPA buffer (50‍ ‍mM Tris-Cl [pH 7.4], 150‍ ‍mM NaCl, 5‍ ‍mM EDTA, 1% Nonidet P-40, 1% sodium deoxycholate, 0.1% sodium dodecyl sulfate, 1% aprotinin, 50‍ ‍mM NaF, and 0.1‍ ‍mM Na_3_VO_4_) 48 or 96 h after the mock or SFVmfu infection (at an MOI of 0.34). Similarly, whole cell lysates of uninfected TE671 and TE671/SFVmfu(PI) cells were obtained with RIPA buffer. Lysates were mixed with Laemmli sample buffer supplemented with 2-mercaptoethanol and boiled at 95°C for 5 min. Each boiled sample was electrophoresed on a sodium dodecyl sulfate-polyacrylamide gel and electrically blotted onto a polyvinylidene difluoride membrane. Each blotted membrane was blocked in 5% milk/Tris-buffered saline with Tween 20 and incubated with an anti-CAP1 antibody (Abcam [catalog number: EPR8339{B}]) as the primary antibody or a goat anti-rabbit IgG-horseradish peroxidase antibody (Thermo Fisher Scientific [catalog number: 31466]) as the secondary antibody. Antibody reactions were detected and processed with the SuperSignal West Femto system (Thermo Fisher Scientific) using a Luminescent Image Analyzer LAS4000 mini (Fujifilm).

### RISC activity assay

The RISC activity assay was conducted to verify that miRNA expression plasmids produce the intended miRNAs. HEK293T cells in collagen-coated 24-well plates (Iwaki) were co-transfected with 40 ng of each firefly luciferase reporter plasmid, 4 ng of pRL-TK (*Renilla* luciferase reporter plasmid) (Promega), and 456 ng of each expression plasmid, as indicated in Fig. 3B, using Lipofectamine 2000 (Thermo Fisher Scientific) according to the manufacturer’s instructions. Cells were harvested 24 h after transfection and subjected to the luciferase assay with the Dual-Glo Luciferase Assay System (Promega) using Lumat LB9507 (Berthold). The significance of differences was assessed using the Student’s *t*-test.

### Host target 3′UTR luciferase assays

HEK293T cells in collagen-coated 24-well plates (Iwaki) were co-transfected with 5 ng of pGL3-hCMV/CAP1 3′UTR (either Wild-type, Single mut., or Triple mut.), 5 ng of pRL-TK (Promega), and 790 ng of either empty plasmid (pUC18 or pcDNA3.1[+]) or each miRNA expression plasmid, as indicated in Fig. 4B, in triplicate using Lipofectamine 2000 (Thermo Fisher Scientific) according to the manufacturer’s instructions. Cells were harvested 24 h after transfection and subjected to the luciferase assay with the Dual-Glo Luciferase Assay System (Promega) using Lumat LB9507 (Berthold). The significance of differences was assessed using the Student’s *t*-test.

Uninfected TE671 and TE671/SFVmfu(PI) cells in collagen-coated 24-well plates (Iwaki) were co-transfected with 80 ng of pGL3-hCMV/CAP1 3′UTR (either Wild-type, Single mut., or Triple mut.), or pGL3-hCMV, 80 ng of pRL-TK, and 640 ng of the empty plasmid (pcDNA3.1[+]) in triplicate using Lipofectamine 2000 (Thermo Fisher Scientific) according to the manufacturer’s instructions. Cells were harvested 24 h after transfection and assayed for luciferase activity with the Dual-Glo Luciferase Assay System (Promega) using Lumat LB9507 (Berthold). The significance of differences was assessed using the Student’s *t*-test.

### Accession numbers

Illumina Hiseq sequencing data and nucleotide sequences identified in the present study were deposited to the database of the DNA Data Bank of Japan with the following accession numbers: DRA008912, DRA008923, and LC498639.

## Results

### TE671 cells persistently infected with SFVmfu express viral miRNAs

**Fig. 1.**
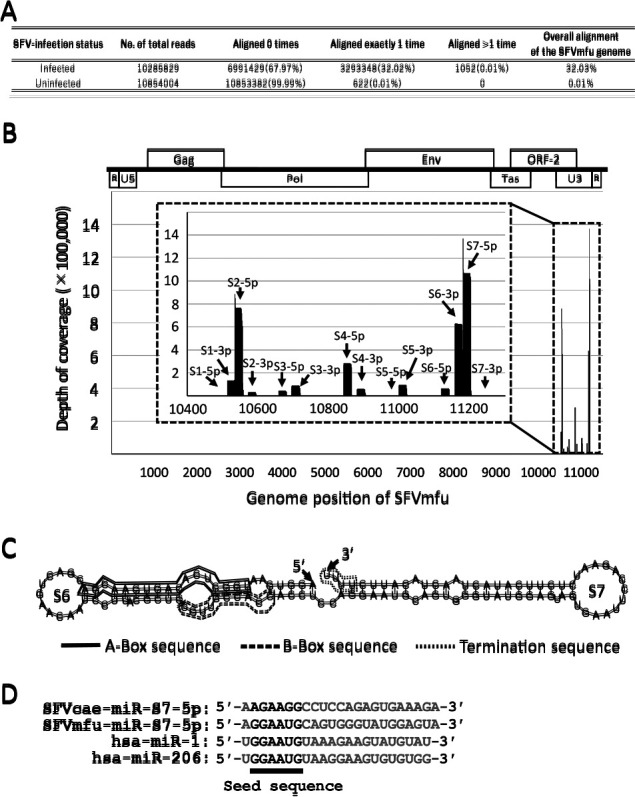
Persistent infection by SFVmfu leads to the expression of miRNAs at a high level. **(A)** Mapping of small RNAs in TE671 and TE671/SFVmfu(PI) cells to the genomes of SFVmfu and humans. **(B)** Small RNA profiling of TE671/SFVmfu(PI) cells. The diagram above the graph shows the location of SFV genes and segments of LTR used for mapping. Fourteen types of mature miRNAs are indicated with arrows. **(C)** Predicted secondary structure of SFVmfu-mir-S6-7 identified in this study. Circles with solid, dashed, and dotted lines indicate the predicted A-box, B-box, and termination sequences, respectively. **(D)** Comparison of seed sequences of miR-S7-5p of SFVmfu and SFVcae with the miR-1 miRNA precursor family (hsa-miR-1 and hsa-miR-206).

Small RNA sequencing analyses were performed using total RNAs extracted from both TE671 and TE671/SFVmfu(PI) cells. Almost all short reads (99.99%) obtained from uninfected TE671 cells were not mapped to the viral genome of SFVmfu, whereas approximately one-third of short reads (32.02%) obtained from TE671/SFVmfu(PI) cells were mapped to the viral genome of SFVmfu (Fig. 1A). As reported previously in SFVcae (Kincaid *et al.*, 2014), seven pre-miRNAs (S1–S7) and 14 mature-miRNAs were identified in SFVmfu, all of which were derived from the U3 region of LTR (Fig. 1B and S1, and Table 1). Moreover, the pre-miRNAs identified contained A/B-box sequences, which are the essential promoter elements for initiating transcription driven by RNA polymerase III (Fig. 1C and S1) (Kassavetis and Geiduschek, 2006). These promoter elements were also identified in the pre-miRNAs of SFVcae (Kincaid *et al.*, 2014). Among the 14 mature miRNAs identified, SFVmfu-miR-S7-5p, which showed the highest expression level, was derived from a dumbbell-shaped pre-miRNA, SFVmfu-mir-S6-7 (Fig. 1C and Table 1). The seed sequence of SFVmfu-miR-S7-5p (“GGAAUG”) was different from that of SFVcae-miR-S7-5p (“AGAAGG”) (Fig. 1D). Notably, the miR-1 miRNA precursor family of the host had the same seed sequence as SFVmfu-miR-S7-5p (Fig. 1D). These results indicate that SFVmfu-miR-S7-5p shares the same target genes as human miR-1 (hsa-miR-1).

### CAP1 is down-regulated by SFVmfu infection in TE671 cells

**Fig. 2.**
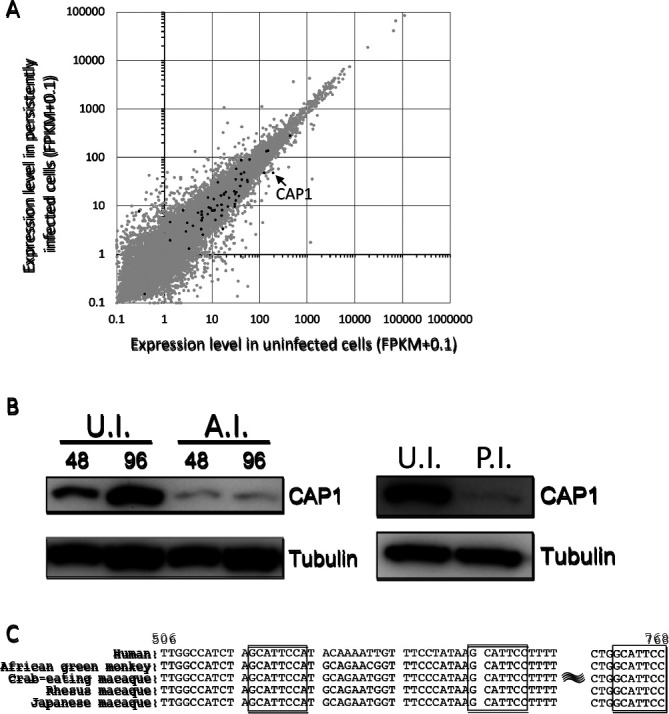
Reduction in the expression of CAP1 by an infection with SFVmfu in TE671 cells. **(A)** Total RNAs of uninfected TE671 and TE671/SFVmfu(PI) cells were extracted and subjected to transcriptome analyses. The gray and black dots indicate the genes that were predicted to have low and high affinities with SFVmfu-miR-S7-5p, respectively (see also Table S2.). **(B)** Immunoblot analyses to detect CAP1. TE671 cells that were either acutely (left panel) or persistently (right panel) infected with SFVmfu were subjected to immunoblot analyses. The numbers on the lanes in the left panel indicate hours post-infection. Abbreviations: U.I.: uninfected TE671 cells; A.I.: TE671 cells acutely infected with SFVmfu; P.I.: TE671/SFVmfu(PI) cells. **(C)** Three predicted recognition sites for SFVmfu-miR-S7-5p present in CAP1 3′UTR from five different primate species. The numbers above the alignment indicate the locations of CAP1 3′UTR in the human genome.

To clarify the function of SFVmfu-miR-S7-5p, a transcriptome analysis was performed in both TE671 and TE671/SFVmfu(PI) cells (Fig. 2A). Among the genes predicted to be recognized by SFVmfu-miR-S7-5p (highlighted with black dots in Fig. 2A), the expression level of CAP1 was relatively high in uninfected TE671 cells, but low in TE671/SFVmfu(PI) cells (app. 75% reduction of FPKM). The immunoblot analysis also indicated that the expression of CAP1 was severely down-regulated at the protein level by both acute and persistent infections with SFVmfu (Fig. 2B). The 3′UTR of CAP1 contained three potential SFVmfu-miR-S7-5p recognition sites, which were conserved across several primate species, including the Japanese macaque and humans (Fig. 2C).

### SFVmfu-miR-S7-5p down-regulates a reporter gene with its complementary sequence in 3′UTR

**Fig. 3.**
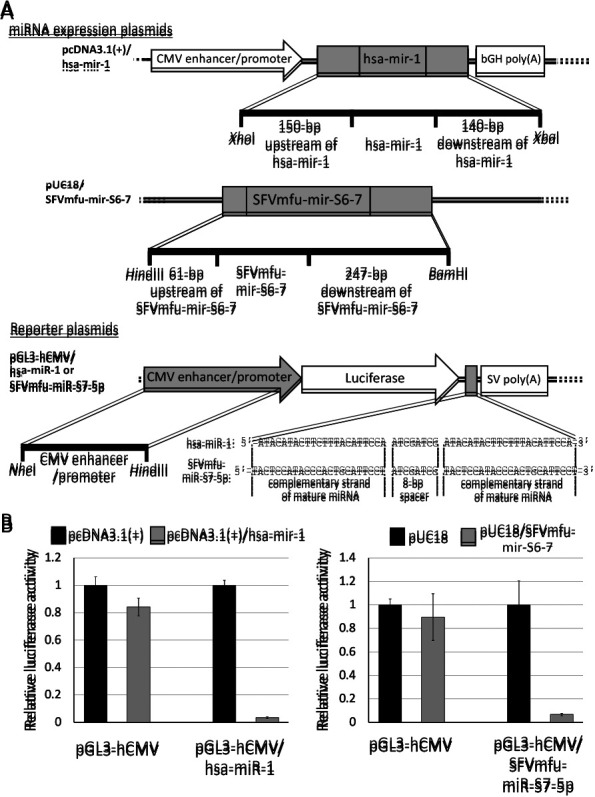
RISC activity assays for miRNA expression plasmids. **(A)** Expression plasmids constructed for hsa-mir-1 (top) and SFVmfu-mir-S6-7 (center), and reporter plasmids constructed for hsa-miR-1 and SFVmfu-miR-S7-5p (bottom). The backbone plasmid (pUC18) for SFVmfu-mir-R6-7 does not contain mammalian promoters; however, the pre-miRNA sequence contains the internal promoter elements (A/B-box sequences). The reporter plasmids contain complementary sequences of mature miRNAs between the firefly luciferase gene and poly**(A)** signal of the pGL3 vector inserted with the hCMV enhancer/promoter. Sections cloned into the backbone plasmids are colored in grey. **(B)** RISC activity assays using the expression plasmids of hsa-miR-1 (left panel) and SFVmfu-miR-S7-5p (right panel). Abbreviations: bGH: bovine growth hormone; SV: simian virus 40.

The RISC activity assay was performed to investigate whether the expression plasmids constructed for SFVmfu-mir-S7-5p and hsa-mir-1 (pUC18/SFVmfu-mir-S6-7 and pcDNA3.1[+]/hsa-mir-1, respectively) produce target-specific miRNAs. A small part of the U3 region of LTR in SFVmfu was used to construct pUC18/SFVmfu-mir-S6-7 (containing the sequence of SFVmfu-miR-S7-5p) in order to avoid overlaps with other viral miRNAs (Fig. 3A [center]). Reporter plasmids were also constructed, which contained the complementary sequences of hsa-miR-1 and SFVmfu-miR-S7-5p downstream of the firefly luciferase gene (Fig. 3A [bottom]). The results obtained showed that both miRNA expression plasmids down-regulated the expression of the reporter plasmids with complementary sequences (Fig. 3B), indicating that both expression plasmids produced functional miRNAs (hsa-miR-1 and SFVmfu-miR-S7-5p, respectively), which silenced the mRNAs containing complementary sequences in 3′UTR.

### SFVmfu-miR-S7-5p suppresses CAP1 expression via recognition sites in 3′UTR

**Fig. 4.**
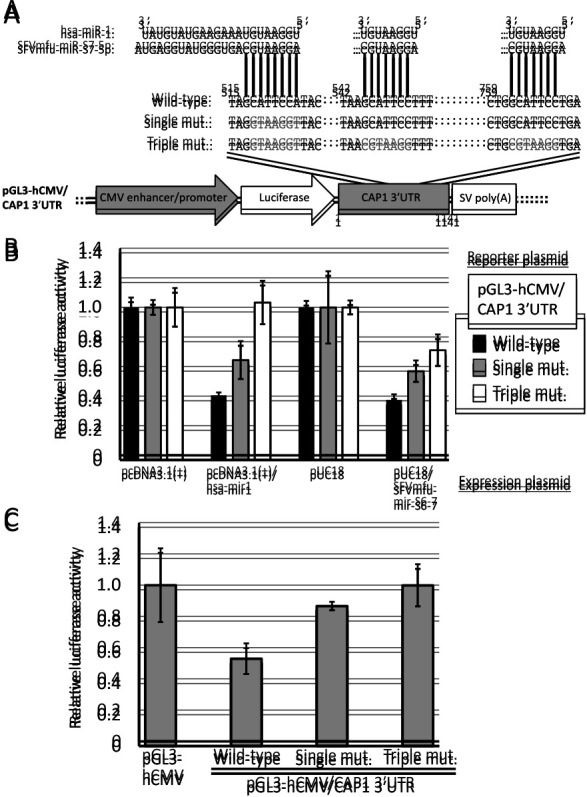
Suppression of CAP1 expression by SFVmfu-miR-S7-5p via recognition sites. **(A)** Reporter plasmids containing the 3′UTR of the CAP1 gene. Nucleotides shown in grey characters indicate mutated miRNA recognition sites. The numbers above the alignment indicate the locations of CAP1 3′UTR in the human genome. **(B)** A luciferase reporter assay was performed by the co-transfection of miRNA expression plasmids with reporter plasmids. **(C)** A luciferase reporter assay was performed using TE671/SFVmfu(PI) cells without miRNA expression plasmids.

To investigate whether SFVmfu-miR-S7-5p recognizes and down-regulates the expression of a gene bearing the 3′UTR of CAP1, we constructed a series of reporter plasmids containing either the wild-type or modified 3′UTR of CAP1 downstream of the firefly luciferase gene (Fig. 4A). hsa-miR-1 and SFVmfu-miR-S7-5p both repressed the expression of luciferase when the reporter plasmids contained the wild-type 3′UTR of CAP1 (Fig. 4B). Luciferase expression levels were restored when the predicted recognition sites were modified (Fig. 4B), suggesting that hsa-miR-1 and SFVmfu-miR-S7-5p both repress CAP1 expression via the recognition sites.

An additional reporter assay was performed on TE671/SFVmfu(PI) cells without miRNA expression plasmids. In TE671/SFVmfu(PI) cells, the level of luciferase expression was suppressed when the reporter plasmid contained the wild-type 3′UTR of CAP1 and was restored when the recognition sites were disrupted (Fig. 4C). Based on these results, we concluded that TE671/SFVmfu(PI) cells express SFVmfu-miR-S7-5p, which is capable of down-regulating CAP1 expression via recognition sites.

## Discussion

In the present study, we showed that persistent infection with SFVmfu in TE671 cells increased the expression levels of viral miRNAs encoded in the U3 region of SFVmfu LTR, as previously reported in SFVcae (Kincaid *et al.*, 2014) (Fig. 1). Among the miRNAs identified, we focused our analyses on SFVmfu-miR-S7-5p, which had the highest read count among the 14 viral miRNAs identified (Fig. 1B). A multiple sequence alignment analysis of SFVs from *Macaca* species indicated that the U3 region of SFVmfu was distinct, but similar to those of SFVs from other macaques (rhesus and Taiwanese macaques [SFVmmu strain DPZ9524 and SFVmcy strain FV34, respectively]) (Fig. S2). Notably, the seed sequence (“GGAAUG”) of miR-S7-5p of SFVmfu was the same as those of SFVmmu and SFVmcy, suggesting that they also have the ability to express miRNA harboring the same seed sequence as SFVmfu-miR-S7-5p. On the other hand, the seed sequence (“AGAAGG”) of SFVcae-miR-S7-5p was markedly different from that of SFVmfu-miR-S7-5p and only moderately expressed (Kincaid *et al.*, 2014). Instead, cells infected with SFVcae expressed SFVcae-miR-S6-3p at a high level, which has the same seed sequence (“AACAGUC”) as hsa-miR-132 (Kincaid *et al.*, 2014). Moreover, SFVcae-miR-S6-3p and hsa-miR-132 both suppressed the expression of EP300 (Kincaid *et al.*, 2014), which is the transcriptional coactivator of the type-1 interferon response (Lagos *et al.*, 2010). However, cells infected with SFVmfu expressed SFVmfu-miR-S6-3p with a different seed sequence (“CGCUGCG”) only at a moderate level (Fig. 1B and S2). Our transcriptome analyses indicated that the expression of EP300 was not significantly changed by the SFVmfu infection (data not shown). These results suggest that the target genes regulated by miRNAs differ between SFVmfu and SFVcae.

The expression of CAP1 was severely down-regulated by the SFVmfu infection in TE671 cells (Fig. 2). In the luciferase reporter assay using miRNA expression plasmids, the luciferase activity of the reporter plasmid containing CAP1 3′UTR was not fully recovered by the disruption of all three predicted miRNA recognition sites (Fig. 4B). On the other hand, when the reporter plasmids were transfected into TE671/SFVmfu(PI) cells, luciferase activity was fully recovered by the disruption of the three recognition sites (Fig. 4C). One possible explanation for this discrepancy is that the use of the plasmid (pUC18) without mammalian promoters may cause the non-specific suppression of the firefly luciferase protein produced from pGL3-based reporter plasmids. This incomplete recovery associated with using a plasmid without mammalian promoters in the luciferase reporter assay was also observed in a previous study (Kincaid *et al.*, 2014). Nonetheless, the complete recovery of relative luciferase activity, in the reporter assay using TE671/SFVmfu(PI) cells, by the disruption of the three recognition sites indicates that SFVmfu-miR-S7-5p down-regulated CAP1 through the predicted recognition sites in cells.

Physiologically, CAP1 is involved in actin cytoskeleton remodeling in mammalian cells, and the knockdown of CAP1 results in impaired cell migration with decreased actin depolymerization (Bertling *et al.*, 2004). Actin depolymerization plays a crucial role in the replication of retroviruses, such as human immunodeficiency virus 1 (Yoder *et al.*, 2008; Gladnikoff *et al.*, 2009; Santos *et al.*, 2012; Wen *et al.*, 2014). However, it currently remains unclear whether the down-regulation of CAP1 by SFVmfu-miR-S7-5p benefits the replication strategy of SFVmfu.

One possible hypothesis for the significance of SFV miRNAs is that viral miRNAs contribute to the autoinhibitory regulation of SFV gene expression in infected cells. The deletion of the 5′-portion of the U3 region (a possible miRNA cluster) in a chimpanzee-derived SFV was shown to increase viral replication in fibroblasts (Schmidt *et al.*, 1997). SFV may utilize miRNAs to optimize long-term co-existence with the host, as reported for other viruses, such as herpes simplex virus 1 and polyomavirus (Umbach *et al.*, 2008; Broekema and Imperiale, 2013).

Another hypothesis for the significance of SFV miRNAs is that the miRNAs of SFVs suppress the expression of genes that are involved in the development of cancers. Among the family *Retroviridae*, gammaretroviruses preferentially integrate into the vicinity of the TSSs of the host genes (Desfarges and Ciuffi, 2010). This integration potentially leads to tumorigenesis when integration occurs in the promoter/enhancer regions of proto-oncogenes. Similar to gammaretroviruses, FVs are also more likely to integrate into the vicinity of TSSs (Trobridge *et al.*, 2006). However, the development of cancer by SFV infection has not yet been reported. Thus, SFVs may utilize as-yet-unknown mechanisms to avoid the development of cancer in host primates. SFVmfu-miR-S7-5p may counter the development of tumors by repressing the expression of CAP1 in infected cells. It is interesting to note that the expression level of hsa-miR-1, which shares a seed sequence with SFVmfu-miR-S7-5p, was down-regulated in various types of cancers (Liu *et al.*, 2015; Han *et al.*, 2017; Korde *et al.*, 2017). The up-regulation of CAP1 has been observed in several solid tumors, such as ovarian cancer, breast cancer (Xie *et al.*, 2014; 2018; Hua *et al.*, 2015; Hasan and Zhou, 2019), and rhabdomyosarcoma from which the TE671 cell line is derived (Rao *et al.*, 2010). Rhabdomyosarcoma is also known to have a reduced expression level of hsa-miR-1, which suppresses CAP1 expression (Vinther *et al.*, 2006; Rao *et al.*, 2010). Interestingly, SFVcae-miR-S6-3p shares a seed sequence with hsa-miR-132, which is also down-regulated in several types of tumors (Formosa *et al.*, 2012; Wang *et al.*, 2014; Zhang *et al.*, 2016). SFVs may utilize distinct miRNAs to avoid tumorigenesis by suppressing various onco-related genes.

## Supplementary Material

Supplementary Material

## Figures and Tables

**Table 1.** List of mature miRNA sequences identified in this study

Name	Sequence (5′-3′)	Length (nt)	Shape of pre-miRNA	Start position	End position	Number of reads
SFVmfu-miR-S1-5p	AGGAAGCAUUUGGUAAAUUCUAC	23	Dumbbell shape with S2	10,476	10,498	121
SFVmfu-miR-S1-3p	UCAUUUACUACCGUGCUUCCGA	22		10,512	10,533	135,125
SFVmfu-miR-S2-5p	UGGAGAACUUAGGGACGAGGCU	22	Dumbbell shape with S1	10,534	10,555	655,144
SFVmfu-miR-S2-3p	UCCUCAUCUCGAGUGUCUCCCUUU	24		10,571	10,594	20,786
SFVmfu-miR-S3-5p	AAGGGAGGGAGUGGAACGUCCUG	23	Single hairpin	10,658	10,680	35,207
SFVmfu-miR-S3-3p	UGACGCUCUCCCAUCCCUCCUUUUC	25		10,695	10,719	67,292
SFVmfu-miR-S4-5p	UCAAGAACCCAGGGAGCAAUGUUG	24	Single hairpin	10,842	10,865	250,036
SFVmfu-miR-S4-3p	AUAGUGCAUCCUGGUCGUUCUUUA	24		10,881	10,904	55,352
SFVmfu-miR-S5-5p	ACAAAGGGAAAUAGCUAAUGUGCA	24	Single hairpin	10,961	10,984	44
SFVmfu-miR-S5-3p	UACUUAGCCGUUCUCCUUUGAUA	23		10,999	11,021	91,370
SFVmfu-miR-S6-5p	AGGUGAAUGGCUCACAGUGAACGA	24	Dumbbell shape with S7	11,120	11,143	57,461
SFVmfu-miR-S6-3p	UACGCUGCGUUGCCACCACCU	21		11,159	11,179	620,914
SFVmfu-miR-S7-5p	AGGAAUGCAGUGGGUAUGGAGUA	23	Dumbbell shape with S6	11,182	11,204	1,014,472
SFVmfu-miR-S7-3p	UUCAUACUAACUACAUUCUUUU	22		11,222	11,243	434
