# Supplementary material for: Persistent Infection of Simian Foamy Virus Derived from the Japanese Macaque Leads to the High-Level Expression of microRNA that Resembles the miR-1 microRNA Precursor Family
Source: Microbes Environ. 2020 Jan 23;35(1):ME19130. doi: 10.1264/jsme2.ME19130 (PMC7104284; doi:10.1264/jsme2.ME19130)
Supplement: Supplementary file 1 — Supplementary Material [file 35_19130_s1.pdf]

Supplementary materials

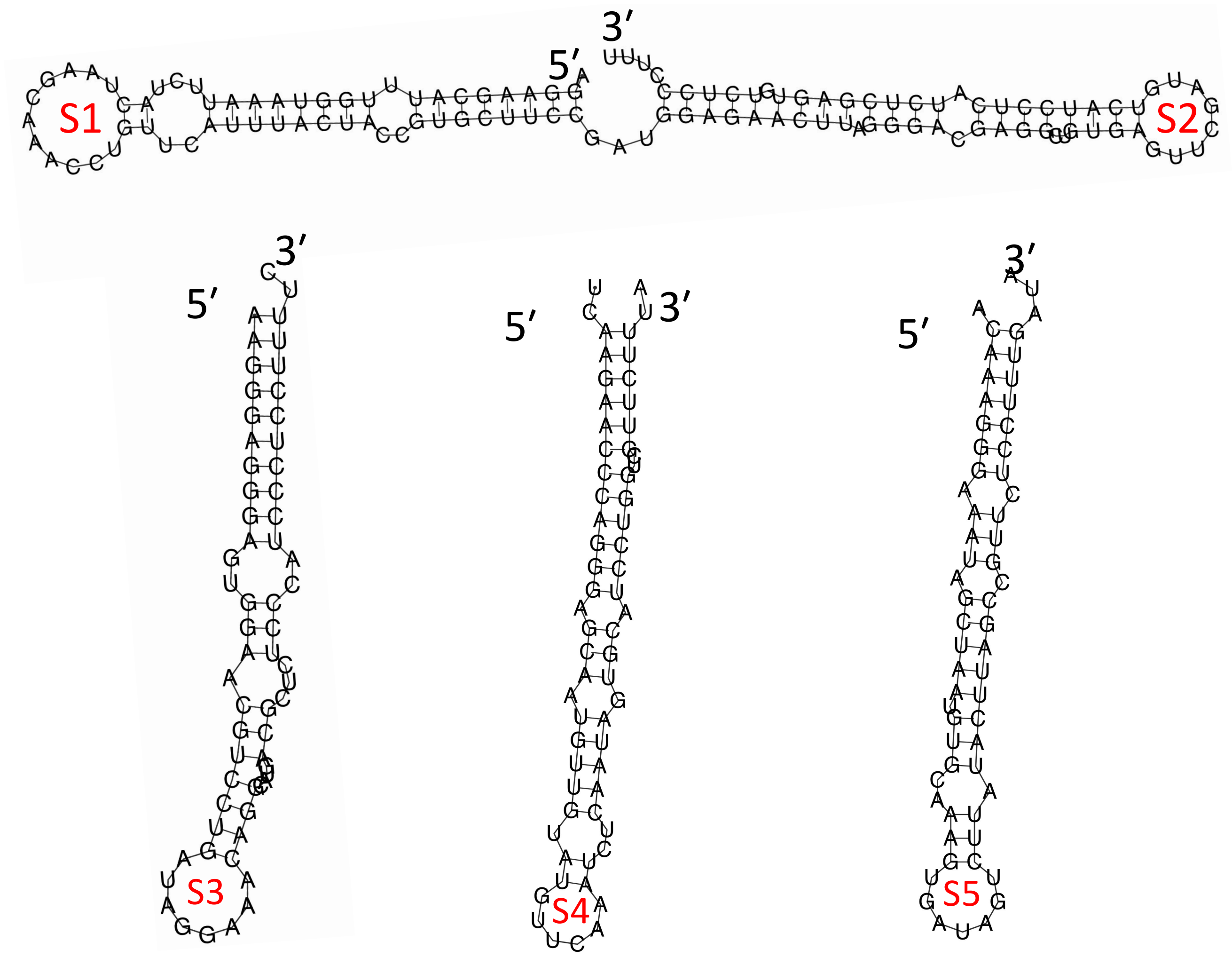

Fig. S1. The predicted secondary structures of pre-miRNA sequences of S1-2, S3, S4, and S5 identified in this study.

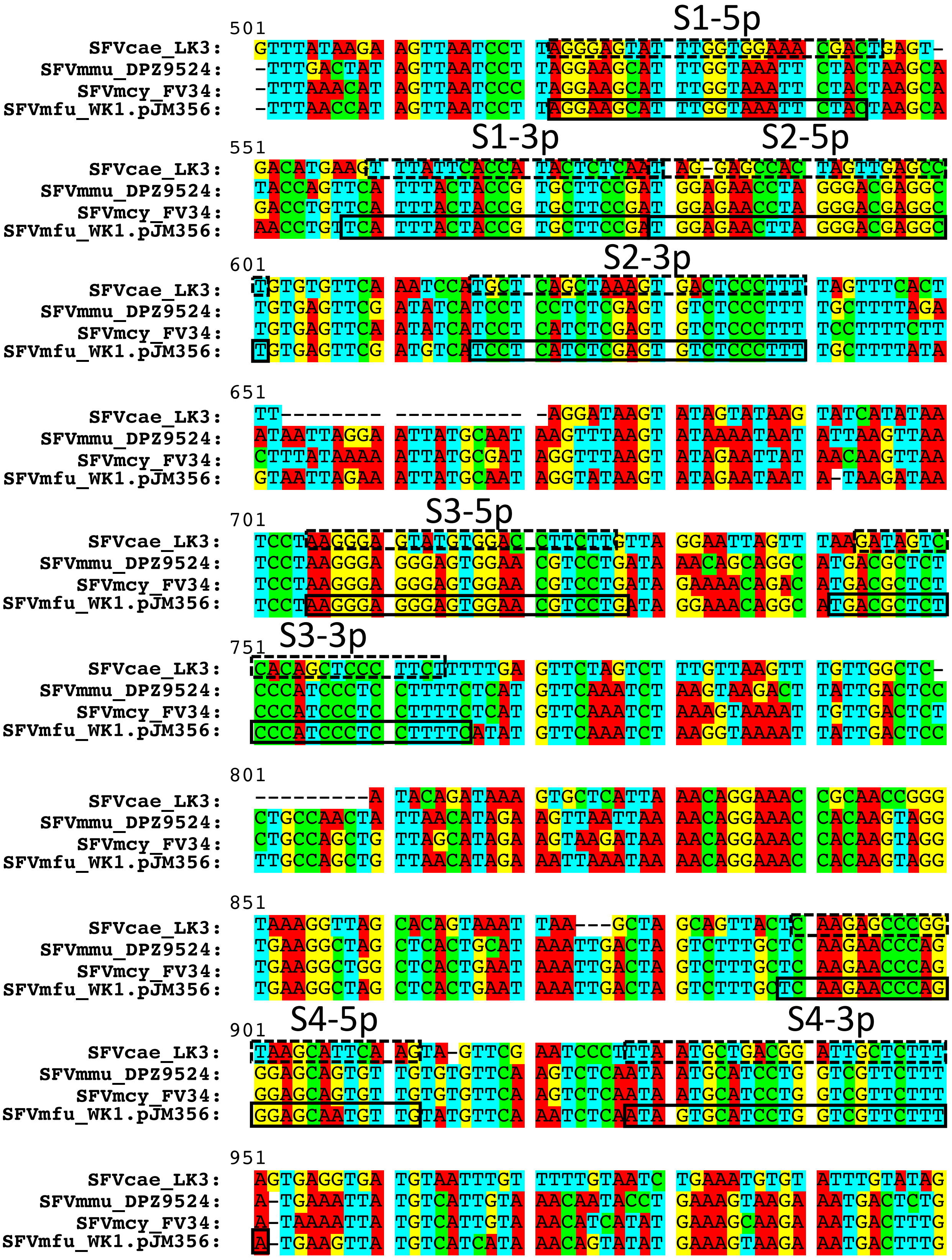

Fig. S2. The multiple sequence alignment of LTRs from SFVs from four primate species. The locations where the miRNAs were expressed are boxed with dotted and solid lines for SFVcae and SFVmfu, respectively.

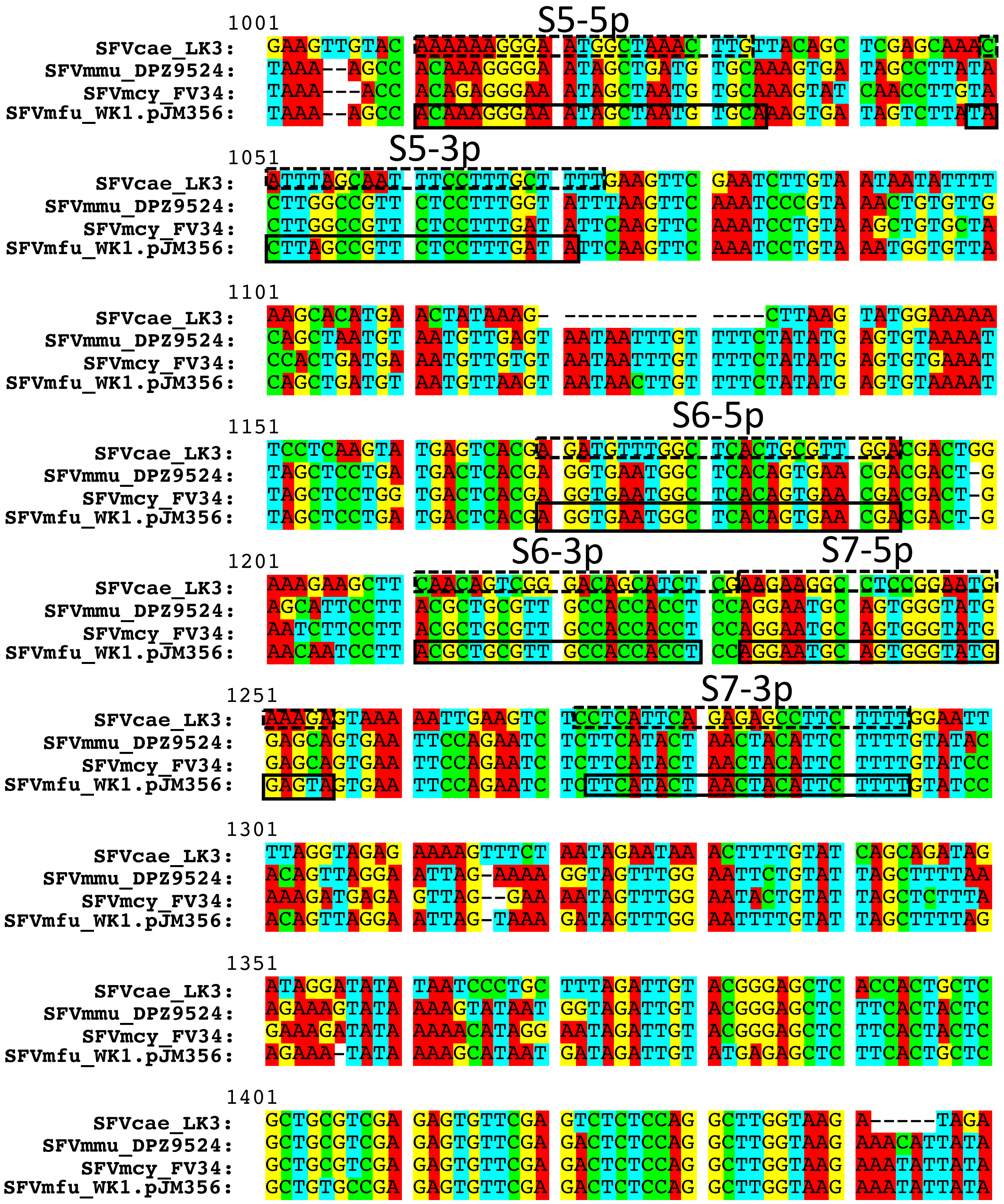

Fig. S2. (continued)

Table S1. Primers used in this study

Name	Purpose of PCR	Template	Target sequence	Sequence (5' - 3')
SFVmiRNA_S67_F	To construct miRNA expression plasmids	genomic DNA of TE671 cells infected with SFVmfu	pre-miRNA sequence of miRNA SFVmfu S6-7	CCCAAGCTTGTAATGTTAAGTAATAAGTTG
SFVmiRNA_S67_R				CGCGGATCCGCAGAGCATTAAAAGGATCAGAA
hsa-mir-1-F		genomic DNA of TE671 cells	pre-miRNA sequence of miRNA 1	ATCGCTCGAGGGCTGTCCTGCTCACACAGA
hsa-mir-1-R				CGATTCTAGAGCGGGGACACGACCGTCCAC
hCMVenh-Nhe1_F		pcDNA 3.1(+)	CMV enhancer and promoter	GCGAGCTAGCCAGATATACGCGTTGACATTG
hCMVpro-Hind3_R				TTAGAAGCTTAGCTCTGCTTATATAGACCTCCCA
miR1_comp_F	To construct luciferase reporter plasmids	No template (annealing and extending primers)	Complementary sequences of miRNA-1	GATCGCCGTGTAATTATACATACTTCTTTACATTCCAATCGATCGATACATACTTC
miR1_comp_R				CCGGCCGCCCCGACTTGGAATGTAAAGAAGTATGTATCGATCGATTGGAATGTAAA
SFV_S7-5'_comp_F		No template (annealing and extending primers)	Complementary sequences of miRNA SFVmfu S7-5p	GATCGCCGTGTAATTTACTCCATACCCACTGCATTCCTATCGATCGTACTC
SFV_S7-5'_comp_R				CCGGCCGCCCCGACTAGGAATGCAGTGGGTATGGAGTACGATCGATAGGAATG
CAP1_pGL3_3UTR_F		genomic DNA of TE671 cells	3'UTR of human CAP1 gene	GATCGCCGTGTAATTGCGAAGTGCCACTGGGTTC
CAP1_pGL3_3UTR_R				CCGGCCGCCCCGACTGCAAGTTTGGTATTAACTTTATTC
CAP1mut_F		pGL3-hCMV/CAP1 3'UTR (Wild-type)	3'UTR of human CAP1 gene	CTAGCAGTAAGGTTACAAAATTGTTTCC
CAP1mut_R				TGTAACCTTACCTAGATGGCCAAGCCTCAG
CAP1mut_2nd_F		pGL3-hCMV/CAP1 3'UTR (Single mut.)	3'UTR of human CAP1 gene	CTATAACGTAAGGTTTTATTCTCTATTC
CAP1mut_2nd_R				AAAACCTTACGTTATAGGAAACAATTTTG
CAP1mut_3rd_F		pGL3-hCMV/CAP1 3'UTR (Double mut.)	3'UTR of human CAP1 gene	AGTCTGCGTAAGGTGAATCCTTCTTCCC
CAP1mut_3rd_R				ATTCACCTTACGCAGACTCCTTGAAATC
Puro-InSIN_F	To obtain puromycin resistant gene	pMX-puro	puromycin resistant gene	CAGGATGAGGATCGTACCATGACCGAGTACAAGCCC
Puro-InSIN_R				GATAGAAGGCGATGCTCAGGCACCGGGCTTGCGG
CAP1-F	To sequence 3'UTR of CAP1 from Japanese macaque	Genomic DNA of Japanese macaque	3'UTR of CAP1 from Japanese macaque	CGCGAAGCTTGTTCAAGACCCTCTGGAATGGGCAG
CAP1-R				GGCAGAATTCGGATGTGAAAATAATTTTATGAGGT

Table S2. Predicted target genes of SFVmfu-miR-S7-5p obtained from miRDB (accessed in August 2019)

Target Rank	Gene Symbol	Target Score	Gene Description
1	MMD	100	monocyte to macrophage differentiation associated
2	CAP1	100	cyclase associated actin cytoskeleton regulatory protein 1
3	PTPRD	99	protein tyrosine phosphatase, receptor type D
4	SYT1	99	synaptotagmin 1
5	MCTP1	99	multiple C2 and transmembrane domain containing 1
6	CRIM1	98	cysteine rich transmembrane BMP regulator 1
7	GPR158	98	G protein-coupled receptor 158
8	SGK1	98	serum/glucocorticoid regulated kinase 1
9	XPO6	97	exportin 6
10	XPNPEP3	97	X-prolyl aminopeptidase 3
11	GLCCI1	97	glucocorticoid induced 1
12	ZFP36L2	97	ZFP36 ring finger protein like 2
13	USP46	97	ubiquitin specific peptidase 46
14	SMIM14	97	small integral membrane protein 14
15	SLC39A10	97	solute carrier family 39 member 10
16	EML4	97	EMAP like 4
17	AP3S1	97	adaptor related protein complex 3 subunit sigma 1
18	COL25A1	97	collagen type XXV alpha 1 chain
19	GJA1	97	gap junction protein alpha 1
20	GOLPH3	96	golgi phosphoprotein 3
21	NOTCH3	96	notch 3
22	PDCD4	96	programmed cell death 4
23	MTX1	96	metaxin 1
24	PABPC1	95	poly(A) binding protein cytoplasmic 1
25	KAT6A	95	lysine acetyltransferase 6A
26	WDR47	95	WD repeat domain 47
27	NCOA1	95	nuclear receptor coactivator 1
28	SS18	95	SS18, nBAF chromatin remodeling complex subunit
29	GNPTAB	95	N-acetylglucosamine-1-phosphate transferase subunits alpha and beta
30	BTAF1	95	B-TFIID TATA-box binding protein associated factor 1
31	ADAR	95	adenosine deaminase, RNA specific
32	ZNF281	94	zinc finger protein 281
33	GCLC	94	glutamate-cysteine ligase catalytic subunit
34	NPHS1	94	NPHS1, nephrin
35	TFE3	94	transcription factor binding to IGHM enhancer 3
36	TMIGD3	94	transmembrane and immunoglobulin domain containing 3
37	GLCE	94	glucuronic acid epimerase
38	ARHGAP32	94	Rho GTPase activating protein 32
39	POGK	93	pogo transposable element derived with KRAB domain
40	C4orf19	93	chromosome 4 open reading frame 19
41	PTPN14	93	protein tyrosine phosphatase, non-receptor type 14
42	HP1BP3	93	heterochromatin protein 1 binding protein 3
43	SLC44A2	93	solute carrier family 44 member 2
44	ARF4	93	ADP ribosylation factor 4
45	SLC44A1	93	solute carrier family 44 member 1
46	TSHR	93	thyroid stimulating hormone receptor
47	SLC10A7	93	solute carrier family 10 member 7
48	LRCH1	92	leucine rich repeats and calponin homology domain containing 1
49	PDIK1L	92	PDLIM1 interacting kinase 1 like
50	FAM102A	92	family with sequence similarity 102 member A
51	KDELR2	92	KDEL endoplasmic reticulum protein retention receptor 2
52	PDLIM2	92	PDZ and LIM domain 2
53	SEPHS2	92	selenophosphate synthetase 2
54	PPRC1	92	peroxisome proliferator-activated receptor gamma, coactivator-related 1
55	SERP1	92	stress associated endoplasmic reticulum protein 1
56	GNPDA2	91	glucosamine-6-phosphate deaminase 2
57	ATP6V1B2	91	ATPase H+ transporting V1 subunit B2
58	STAG2	91	stromal antigen 2
59	COG3	91	component of oligomeric golgi complex 3
60	NECTIN2	91	nectin cell adhesion molecule 2
61	KLF15	91	Kruppel like factor 15
62	HIGD1A	91	HIG1 hypoxia inducible domain family member 1A
63	HMBOX1	91	homeobox containing 1
64	PGBD5	91	piggyBac transposable element derived 5
65	ZNF326	91	zinc finger protein 326
66	HAND2	91	heart and neural crest derivatives expressed 2
67	ACVR1	90	activin A receptor type 1
68	AFF3	90	AF4/FMR2 family member 3
69	CLTC	90	clathrin heavy chain
70	UNK	90	unk zinc finger
71	TRPC7	90	transient receptor potential cation channel subfamily C member 7
72	C2CD4B	90	C2 calcium dependent domain containing 4B
73	CLCN5	90	chloride voltage-gated channel 5
